# The Biotechnology of *Ugba*, a Nigerian Traditional Fermented Food Condiment

**DOI:** 10.3389/fmicb.2016.01153

**Published:** 2016-08-03

**Authors:** Nurudeen A. Olasupo, Chimezie P. Okorie, Folarin A. Oguntoyinbo

**Affiliations:** ^1^Food Microbiology Research Laboratory, Department of Microbiology, Faculty of Science, Lagos State UniversityLagos, Nigeria; ^2^Department of Biotechnology, Federal Institute of Industrial ResearchLagos, Nigeria; ^3^Department of Microbiology, Faculty of Science, University of LagosLagos, Nigeria

**Keywords:** microbiology, *Ugba*, fermentation, condiment

## Abstract

Legumes and oil bean seeds used for the production of condiments in Africa are inedible in their natural state; they contain some anti-nutritional factors especially undigestible oligosaccharides and phytate. Fermentation impact desirable changes by reducing anti-nutritional factors and increasing digestibility. *Ugba* is an alkaline fermented African oil bean cotyledon (*Pentaclethra macrophylla*) produced by the Ibos and other ethnic groups in southern Nigeria. Seen as a family business in many homes, its preparation is in accordance with handed-down tradition from previous generations and serves as a cheap source of plant protein. Its consumption as a native salad is made possible by fermentation of the cotyledon for 2–5 days, but could also serve as a soup flavoring agent when fermentation last for 6–10 days. The fermentation process involved is usually natural with an attendant issue of product safety, quality and inconsistency. The production of this condiment is on a small scale and the equipment used are very rudimentary, devoid of good manufacturing procedures that call to question the issue of microbial safety. This paper therefore reviews the production process and the spectrum of microbial composition involved during fermentation. In addition, potential spoilage agents, nutritional and biochemical changes during production are examined. Furthermore, information that can support development of starter cultures for controlled fermentation process in order to guarantee microbiological safety, quality and improved shelf life are also discussed.

## Introduction

*Ugba*, a product of alkaline fermentation of oil bean seeds (*Pentaclethra macrophylla*) is very popular among the Ibos and other ethnic groups in southern Nigeria. The product serves both as a delicacy and a food flavoring agent. As an important nutritional item, *ugba* is very rich in protein. It similarly plays an economic, social and cultural role among the Ibos in the eastern part of Nigeria. The production of *ugba* is usually pursued as a family business that has become an art that is handed over from one generation to another.

**Figure 1:**
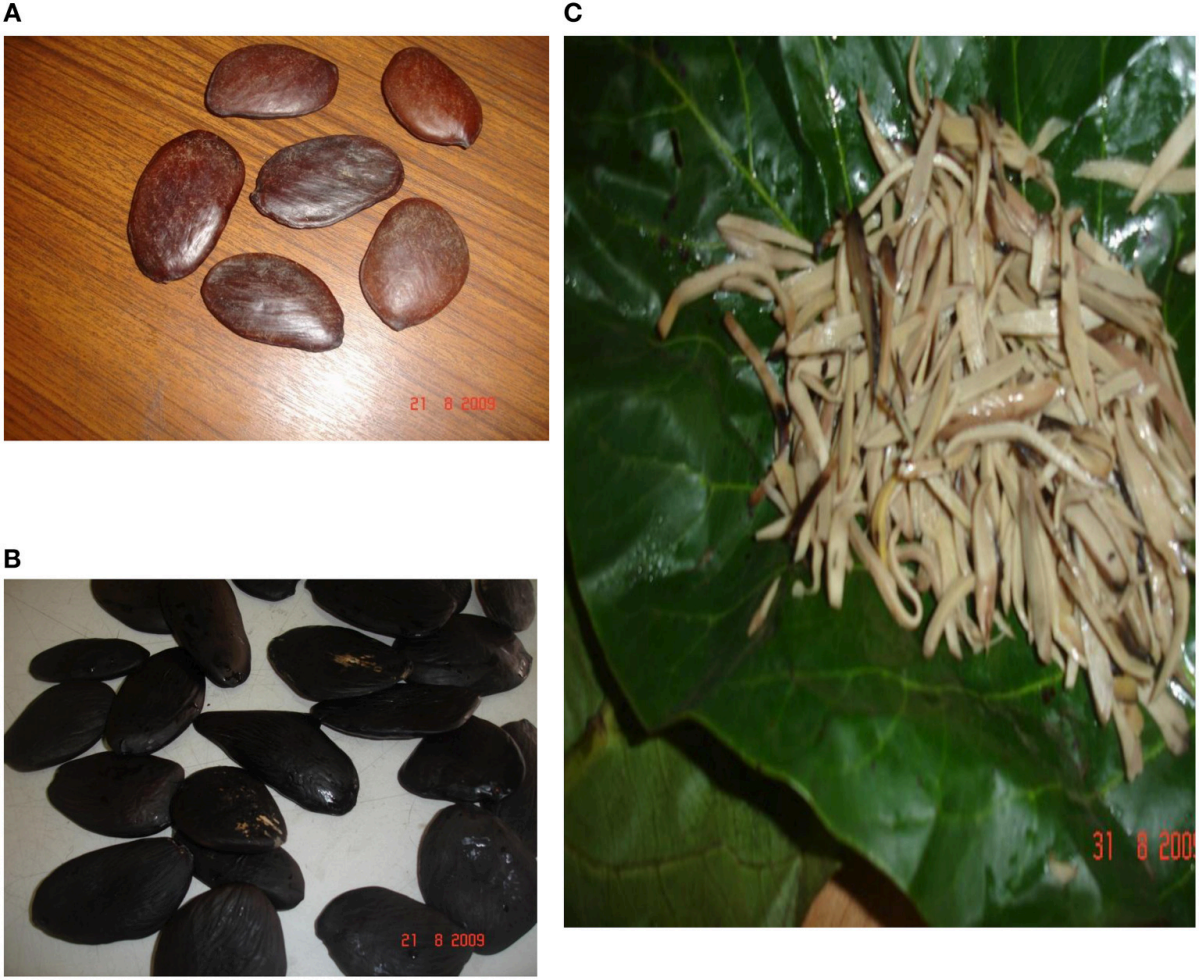
**African oil bean seed (A), Dehulled seeds of African oil bean (B) and Processed slices of the African oil bean cotyledon (C)**. (Okorie and Olasupo, 2013a).

The processing of these large brown glossy seeds of the African oil bean (Figure 1) to obtain *ugba* is usually by natural fermentation, a process that involves microbiological and biochemical changes, caused by hydrolysis and desirable changes. This process is usually influenced by the raw materials and the processing method with variations observed from one production batch or producer to another (Steinkraus, 1983).

Most studies on African fermented foods have focused on isolation and identification of desirable microorganisms involved in the fermentation process. The general consensus from these studies is that fermented African oil bean seeds during *ugba* fermentation is predominantly brought about by bacteria identified as *Bacillus* species (Odunfa, 1981; Obeta, 1983; Isu and Ofuya, 2000; Okorie and Olasupo, 2013a; Eze et al., 2014). Other groups of bacteria have also been implicated in the fermentation of this product and they include species of *Escherichia, Proteus, Micrococcus, Staphylococcus, Streptococcus, Alcaligenes, Pseudomonas, Corynebacterium*, and *Enterococcus* (Oyeyiola, 1981; Odunfa, 1986; Sanni et al., 2002; Okorie and Olasupo, 2013a). No fungi or yeast species have been implicated in the fermentation of *ugba*.

There is very little information on the occurrence and growth of pathogens in African fermented foods. The natural fermentation process used routinely for *ugba* production allows participation of diverse microorganisms. The involvement of pathogenic and spoilage microorganisms during production cannot be totally ruled out, especially if fermentation takes place under very poor hygiene conditions and sanitation, which is a very common occurrence in West Africa. Product inconsistency as a result of mixed-culture processing and post-fermentation contamination constitutes a major challenge to microbial safety and quality of this product.

Production of *ugba* in Nigeria is still on a small scale industrial process involving production at the household level where there is little or no consideration for good manufacturing practices (GMP) and sanitation (Olasupo et al., 2002; Gadaga et al., 2004). Consequently, microbiota responsible for fermentation is often unpredictable and equipment used is rudimentary. Similarly poor hygiene of handlers, lack of portable water and other raw materials often introduce spoilage and pathogenic microorganisms. All these factors affect the quality of the final product and ultimately the health of the consumers. Fermentation period is chosen according to human judgment and varies from one manufacturer to the other. The lack of standardization in the production process often results in product inconsistency and quality variation.

Lactic fermentation is noted to be a major mode of food processing used to achieve preservation and improve shelf life of foods especially in the West African sub-region, where cereals and tubers are processed to variety of foods. This practice has been very reliable in terms of maintaining quality and safety of food especially at the household level where many of the traditional foods are produced (Steinkraus, 1983). Unfortunately, alkaline fermentation of legumes is about hydrolysis of proteins and release of amino acids and ammonia responsible for the pungent smell as well as characteristic flavor. This preservative influence of condiments after fermentation appears to be limited; similar observation has been reported during the processing of fermented African oil bean seeds. The unfermented seeds are much more stable with longer shelf life than the fermented products. Fermentation thus leads to flavor enhancement, complex molecules reduction (oligosaccharides and proteins) but reduces the shelf life of the seeds and exposes the product to post fermentation contamination (Mbajunwa et al., 1998; Oguntoyinbo et al., 2007). Post processing techniques proposed for condiment production in Africa include drying and salting of final product (Achi, 2005; Eman, 2009). However, while these methods could increase shelf life considerably, it is characterized with inherent disadvantages such as loss of volatile compounds and vitamins. Also, the consumption of salt in diet has been identified as having deleterious effects on human health, responsible for cardiovascular diseases in the West African sub-region (Brown et al., 2009; He and MacGregor, 2009; Strazzullo et al., 2009).

Since fermentation of African oil bean seeds increases pH toward alkalinity (pH 8) (Odunfa, 1985a; Sanni and Oguntoyinbo, 2014), the anti-microbial effect often associated with most fermented food due to lowering of pH to acidity is lacking in this product. It is therefore possible that some organisms that are of public health concern could survive the fermentation process. Whether the presence of these organisms is as a result of post-fermentation contamination or they survive the fermentation process, their presence in the product portends great danger to the consuming public. The risk is particularly high also because the product can be eaten without pre-heating. The alkaline pH selects and encourages the dominance of *Bacillus* species. This has been consistently reported to be due to production of peptides, amino acids and ammonia during the hydrolysis of the cotyledons.

Recently, Oguntoyinbo (2014) reported that very little attention is placed on the type of packaging used for many traditional foods in West Africa. Unhygienic and substandard packaging materials can engender easy contamination by hazardous materials, including biological, physical, and chemical hazard of well-prepared foods during preservation. *Ugba* is usually wrapped in leaves (in most cases banana leaf), and nylon bags and sold to the public. These packaging materials could be the source of contamination of the product.

Many of the agricultural raw materials used for the preparation of traditional W. African food products contain endogenous toxins (Kar and Okechukwu, 1978; Okorie and Olasupo, 2014). However, studies have shown that fermentation drastically reduces anti-nutritional factors in many fermented legumes-based foods (Oboh et al., 1998; Khan et al., 2012; Okorie and Olasupo, 2014). It is well known that these foods contain naturally occurring toxins and anti-nutritional compounds. The removal of anti-nutrients from Nigerian fermented food is an important step in ensuring toxicological safety and quality. Fermentation plays significant roles in detoxification of substrates; for instance, removal of toxins during *kawal* production, through the fermentation of the leaves of *Cassia obtusitfolia* in Sudan has been shown to improve safety quality and acceptability (Egwim et al., 2013; Taylor and Duodu, 2015).

Most of the legumes and oil seeds used for the production of condiments are inedible in their unfermented state because they suffer from one drawback or the other. For instance, legumes are a particularly rich source of natural toxicants, including proteinase inhibitors, amylase inhibitors, metal chelates, flatus factors, hemagglutinins, saponins, cyanogens, lathyrogens, tannins, allergens, acetylenic furans, and isoflavonoid phytoalexins (Issoufou et al., 2013; Oguntoyinbo, 2014). The unfermented African oil bean seeds contain a number of anti-nutritional and /or toxic factors including saponins, alkaloids (alkaloid paucine), sterols, glycosides, and growth depressant caffeolyputrescine, but no hemagglutinins (Kar and Okechukwu, 1978).

Understanding the biotechnological principles during fermentation of African oil bean seeds is a crucial strategy for the process optimization of fermented condiments in West Africa. The understanding of the microbiological dynamics, biochemical kinetics and toxicology during fermentation will significantly impact product quality, safety and acceptability. The foregoing has been a review of the different scientific literatures relevant to biotechnology of *ugba* production in Nigeria and highlighted relevant strategies toward process improvement. In addition, current condiment food safety issues are discussed.

## Production process

**Figure 2:**
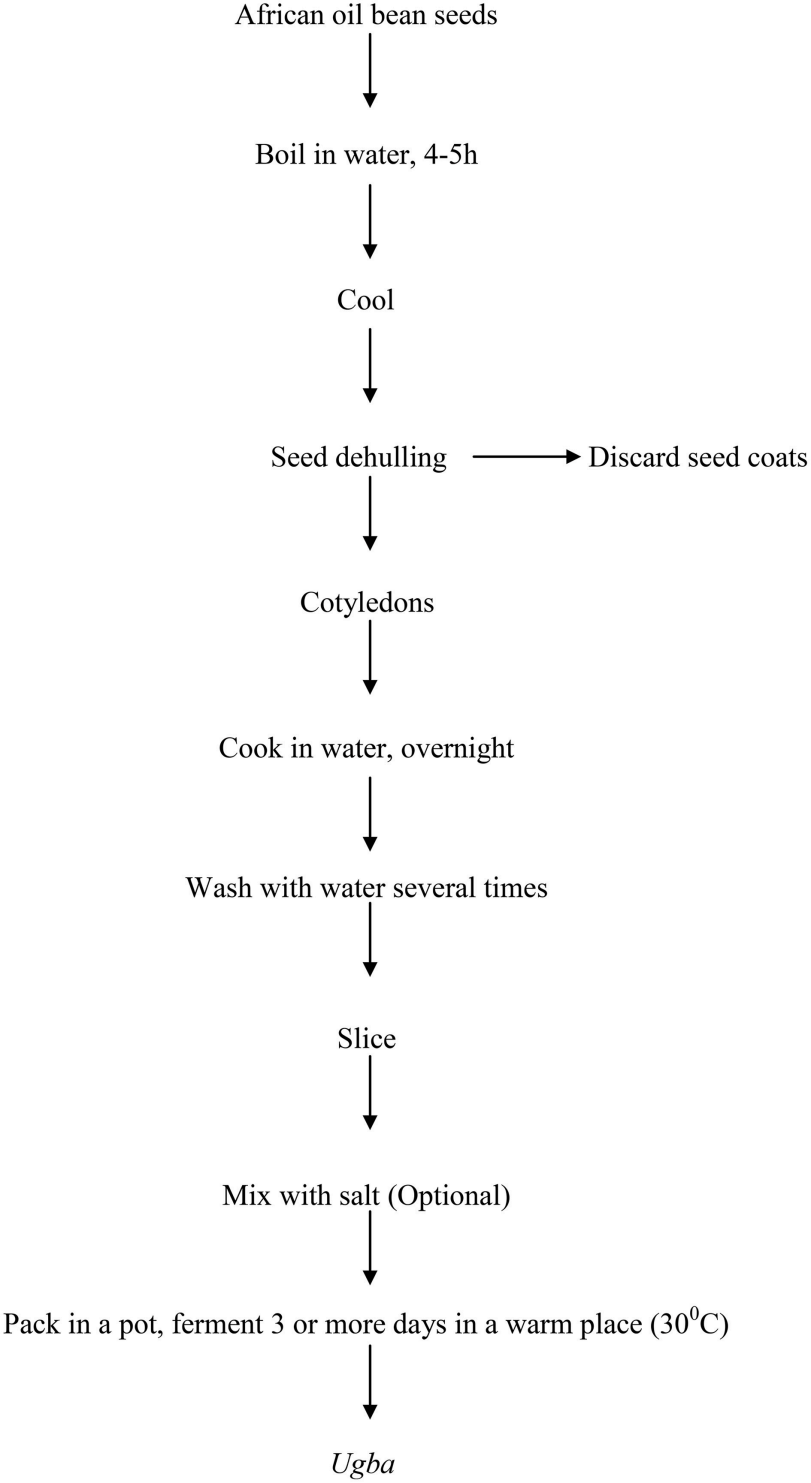
**Flow chart for the preparation of *ugba* (Odunfa and Oyeyiola, 1985)**.

The production process of *ugba* is shown in Figure 2. It has been previously described as alkaline fermentation of the seeds of the African oil bean tree (Ikenebomeh et al., 1986; Sanni et al., 2002; Ogueke et al., 2010). Although the production method varies from one community to the other and from one processor to another, a similar end-product, which usually comes with pungent ammonia-like smell is commonly produced across South Eastern Nigeria (Nwokeleme and Ugwuanyi, 2015). There is variation in boiling time and the procedure that aided dehulling of the seeds. Obeta (1983) reported 16–18 h of boiling, Odunfa and Oyeyiola (1985) and Odunfa (1986), reported initial 12 h boiling time, while Njoku and Okemadu (1989), boiled the seeds for 5–8 h. However, Sokari and Wachukwu (1997) used toasting of the bean seeds in hot (ca. 100°C) sand and holding for a further 30 min at 100°C to dehull the seeds. After dehulling, cotyledons are either sliced or cooked for 30 min or longer. Odunfa and Oyeyiola (1985) reported overnight boiling before soaking and slicing. In the fermentation process, varied methods are used. Odunfa and Oyeyiola (1985) reported that the cotyledons are mixed with salt (sodium chloride ca.1–2 w/w), put in a clean pot, covered and fermented for up to 5 days at room temperature, with or without salt. On the other hand, Sokari and Wachukwu (1997) reported that sliced cotyledons were washed and allowed to drain for ½-1 h, in a basket lined with banana leaves (*Musa sapientum Linn*.) and later wrapped (about 40–50 g of slices per wrap) using another leaf (*Mallotus oppositifolius*) and incubated for 72 h at room temperature.

However, the essential steps in the production of this product are similar and as shown in Figure 2. The differences in the various processing methods described could be responsible for the variations in the products quality observed from one community to the other. The fermented bean slices at the end of the fermentation process are kept near smoldering firewood to develop the characteristic *ugba* flavor and the product is consumed as native salad. However, fermentation for a longer period of time (6–10 days) produces very soft *ugba* which is used as soup flavoring (Odunfa and Oyeyiola, 1985; Sanni et al., 2002). Irrespective of which method is employed in the processing, one major drawback observed is the drudgery involved in the slicing process.

## Microbiological changes during fermentation

The microbiota in fermenting food matrix is a function of the hygienic status of the production environment, the utensil and raw materials used and the handlers. The traditional fermentation method employed in the processing of *ugba* is by chanced inoculation. The microbial interaction in its production is therefore determined by the microbiological status of the raw materials, utensils, handlers and the production environment. Daeschel et al. (1987) and Ling et al. (2013) noted that the dynamics of fermentation in any food matrix is a complex microbiological process involving interactions between different microorganisms. During fermentation of African oil bean seeds, dominant microorganisms capable of enzymatic hydrolysis are responsible for the biochemical and nutritional changes which constitute the observable changes especially in the chemical composition and taste of the final product.

Several works have been carried out on the microbiological changes during fermentation of African oil bean seed for *ugba* production (Obeta, 1983; Odunfa and Oyeyiola, 1985; Ejiofor et al., 1987; Ogueke and Aririatu, 2004; Enujiugha and Akanbi, 2008; Nwagu et al., 2010; Okorie and Olasupo, 2013a). The major fermenting microorganisms involved in the fermentation process have been identified to be proteolytic *Bacillus* species identified as *B. subtilis* (which is the most predominant), *B. licheniformis, B. megaterium, B. macerans*, and *B. circulans* (Obeta, 1983; Sanni, 1993; Isu and Ofuya, 2000; Sanni et al., 2002). The endospores of these bacilli must have been associated with the cotyledons from the beginning of the fermentation. Due to high level of hydrolytic enzyme production by *Bacillus* species, all the species have been reported to have one or more enzymatic hydrolytic properties during legume fermentation (Aderibigbe et al., 1990; Sanni et al., 1999; Oguntoyinbo et al., 2007). However, it appears that *B. subtilis* is the most adapted and dominant with properties such as higher protease and amylase production, production of poly glutamic acid (responsible for mucilage production), pyrazine and antimicrobial such as subtiliosin production (Oguntoyinbo et al., 2007).

Protein has been identified as one of the major components of African oil bean cotyledon (Obeta, 1983). Metabolic and enzymatic hydrolysis of *Bacillus* species serves to break down the protein into amino acids (Isu and Njoku, 1997). Odunfa and Oyewole (1986) and Ghosh et al. (2013) observed that all the *Bacillus* species that have been associated with the fermentation of the oil bean seeds are mainly proteolytic, and 97.3% of these *Bacillus* species are also lipolytic. Proteolysis is therefore the major biochemical activity during the fermentation and has been found to increase constantly during the fermentation of *ugba* and the other food condiments (Odunfa, 1986; Wang and Fung, 1996; Oguntoyinbo et al., 2007). Also, a corresponding increase in the population of *Bacillus* species is reported from the *beginning* of the fermentation process till the end (Ogueke and Aririatu, 2004).

Other groups of organisms that have been found to be associated with the fermentation of this condiment include *Escherichia* species, *Proteus, Pediococcus, Micrococcus, Staphylococcus, Streptococcus, Alcaligenes, Pseudomonas, Corynebacterium, Enterococcus* (Odunfa, 1981; Antai and Ibrahim, 1986; Ogbadu and Okagbue, 1988; Njoku and Okemadu, 1989; Suberu and Akinyanju, 1996; Ogbonna et al., 2001; Okorie and Olasupo, 2013a).

*Staphylococcus* spp. and *Micrococcus* spp. are very active at the early stage of the fermentation process. They rapidly multiply within 24 h of fermentation and then decrease as fermentation progresses. *Escherichia* species, *Proteus* and *Pediococcus* are generally observed to play a minor role in the fermentation process (Odunfa, 1985a) while *Staphylococcus* sp. and *Micrococcus* sp. play a subsidiary role in the production process (Obeta, 1983; Odunfa and Komolafe, 1989).

Apart from proteolysis, other important biochemical changes mediated by microorganisms during the production of this condiment include production of flavor enhancing compounds, production of vitamins and essential fatty acids, and degradation of indigestible oligosaccharides responsible for flatus factors. A reduction in the contents of stachyose, raffinose, and melibiose in fermented soy bean cotyledon during *kinema* production was previously reported (Sarker et al., 1997). Significant increases in thiamine and riboflavin have been observed in *ugba*, and these have been ascribed to riboflavin synthase associated with *Bacillus subtilis* (Odunfa, 1986). These reductions are ascribed to sucrase activities of the *Bacillus* group Aderibigbe and Odunfa (1990) and possibly by the alpha galactosidase activities of the other microorganisms in the fermenting mash, especially *Staphylococcus* sp. and LAB among which alpha galactosidase activities are common (Odunfa and Oyewole, 1998).

Members of the *Enterobacteriaceae* have also been associated with the ecology of fermenting plant proteins (*ugba* inclusive) especially at the early stages of production (Mulyowidarso et al., 1989; Achi, 1992; Okorie and Olasupo, 2013a). These species do not survive until the end of the fermentation, presumably because of the modified environment. It is evident that production of this fermented condiment is initially mediated by a diverse microbial flora, which eventually becomes Gram-positive flora (a reflection of many African fermented foods; Odunfa, 1985b).

## Nutritional changes associated with fermentation of african oil bean seed

Fermentation has been generally observed to improve the nutritional quality of the products obtained. The protein content, essential amino acids, vitamins and mineral contents of most fermented foods have been shown to increase during fermentation.

Fermented foods and beverages harbor diverse microorganisms from the environment, including mycelia molds, yeasts, and bacteria, mostly lactic acid bacteria and micrococci. These microorganisms transform the chemical constituents of raw materials during fermentation and enhance the nutritional value of the products. The activities of these microorganisms are noted to enrich bland diets with improved flavor and texture; preserve perishable foods; fortify products with essential amino acids, bioactive compounds, vitamins, and minerals for healthy living. They also bring about degradation of undesirable compounds and anti- nutritive factors; imparts antioxidant and antimicrobial properties; improve digestibility; and stimulate probiotic functions. While fermentation results in a lower proportion of dry matter in the food product, the concentration of the vitamins, minerals, and protein appear to increase when measured on dry weight basis (Adams, 1990; Chung et al., 2010; Shil et al., 2010; Savadogo et al., 2011; Makanjuola and Ajayi, 2012; Okechukwu et al., 2012; Olakunle and Adebayo, 2012; Tofalo et al., 2012).

African oil bean seeds support diet and improve nutritional availability. Proximate analysis of raw oil bean seed reveals that it is mainly composed of proteins (36–42%), lipids (43–47%) and carbohydrates (4–17%; Odunfa and Oyeyiola, 1985; Njoku and Okemadu, 1989; Ogueke and Aririatu, 2004).

Slight increases in the crude protein and ash contents of the fermented beans have been reported. Enujiugha (2003) reported a steady increase in the level of amino nitrogen from 1.23 mg/Ng-1 DM at the start of fermentation to 13.68 mg/Ng-1 DM after 72 h. The amino acid component of the fermented seed has been shown to contain the 20 essential amino acids (Table 1). The high content of the essential amino acids makes the seed a potential source of protein (Achinewhu, 1982).

**Table 1:** **Amino acid content (g/100 g protein) of African oil bean seeds**.

**Amino acids**	**Content**
Aspartic acid	7.95–10.30
Threonine	3.27–4.17
Serine	4.80–5.54
Glutamic acid	9.32–11.60
Proline	2.90–5.77
Glycine	3.84–4.62
Alanine	3.81–4.70
Cysteine	1.10–4.80
Valine	4.90–6.60
Methionine	0.90–1.80
Isoleucine	3.30–4.88
Leucine	5.30–6.68
Tyrosine	1.80–5.58
Phenylalanine	5.01–7.00
Lysine	5.46–6.97
Histidine	1.53–2.44
Arginine	4.70–6.53
Tryptophan	1.15–1.78

*Source: Mba et al. (1974) and Achinewhu (1982)*.

Glutamic acid appears to be the largest amino acid contained in the seed and its fermented product. This may be responsible for its use as a flavoring agent for soups in south eastern Nigeria. Aspartic acid, lysine and phenylalanine are also present in appreciable amounts in the fermented seeds. In their study of compositional changes in oil bean seeds observed during thermal treatment, Enujiugha and Akanbi (2005) reported a reduction of the protein content from 22.32% dry wt. in the raw seeds to 19.00% dry wt. in the canned product (Table 2). Each processing step brought about a decrease in levels of anti-nutritional factors analyzed. Oxalates, tannins and phytic acid were reduced from 2.79 mg/g, 0.38 g/100 g, and 2.11 g/100 g in the raw seeds to 0.81 mg/g, 0.22 g/100 g, and 1.16 g/100 g in the canned product, respectively.

**Table 2:** **Effect of processing on the proximate chemical composition of African oil bean seeds (mean ± s.d.)**.

**Sample**	**Components (% dry wt)**				
	**Crude protein**	**Oil**	**Crude fiber**	**Ash**	**Carbohydrate**
Raw	22.32 ± 0.37	53.98 ± 0.99	2.13 ± 0.55	2.40 ± 0.11	19.16 ± 0.76
Cooked	19.15 ± 0.13	58.95 ± 0.46	3.26 ± 0.04	1.43 ± 0.13	17.49 ± 0.46
Fermented	17.13 ± 0.21	61.35 ± 1.21	2.93 ± 0.11	1.11 ± 0.04	17.48 ± 1.07
Canned	19.00 ± 0.19	60.11 ± 0.86	3.27 ± 0.12	2.37 ± 0.17	15.26 ± 1.04

*Source: Enujiugha and Akanbi (2005)*.

The oil component of the seed contains about 75% of saturated fatty acids and 25% of unsaturated fatty acids (Kar and Okechukwu, 1978; Table 3). For the saturated fatty acids, lignoceric acid appears to be present in the largest amount constituting about 12% of the total fatty acid concentration, while palmitic acid is the least with 3.4%. The major unsaturated fatty acid in the seeds is linoleic acid constituting 42.8%. Oleic acid is also present in appreciable amounts (29.0%). Linolenic and gadoleic acids are present in very small amounts (3.2 and 0.28%, respectively). The presence of appreciable amounts of behenic and lignoceric acids is not desirable for edible oils (Odunfa, 1986). However, Odoemelam (2005) noted that the high degree of unsaturation makes it suitable for cooking purposes and for use as a drying oil for cosmetics, paints and varnishes.

**Table 3:** Fatty acid composition of African oil bean seeds^*^.

**Composition**	**Values**
Yield of oil (%)	43.3
**SATURATED FATTY ACIDS**	
Palmiitic acid	3.4
Behenic acid	5.2
Lignoceric acid	12.0
**UNSATURATED FATTY ACIDS**	
Oleic acid	29.0
Linoleic acid	42.8
Linolenic acid	3.2
Gadoleic acid	0.28

* *As percentage of total oil*.

*Source: Achinewhu (1982)*.

Fermentation has been found to have minimal effect on the fatty acid content of the oil bean seed. (Onwuliri et al., 2004) reported that fatty acid concentrations did not change appreciably with processing and fermentation. Enujiugha and Akanbi (2005) however observed an increase in the oil content from 53.98 to 60.11%. Information available shows that fatty acid content of the oil bean seeds is not qualitatively affected by fermentation. The principal fatty acid linoleic acid however has been shown to increase from 60.68 to 67.57% of the total fatty acids while oleic acid decreased from 26.95 to 22.59% during fermentation. Palmitic acid and other saturated fatty acids in the seed oil are also slightly affected by fermentation.

Available information shows that the vitamin content of the seeds is low while they are a poor source of calcium and phosphorus (Duke, 1981). The mineral and vitamin contents are observed to decrease during fermentation (Table 4). The niacin and riboflavin of the seeds have been found to decrease during fermentation. Enujiugha and Akanbi (2005) noted that fermentation and canning significantly (*P* < 0.05) reduced the phosphorus and iron contents of the seeds while processing generally raised the calcium and magnesium contents (Table 5).

**Table 4:** **Mineral and vitamin content of unfermented and fermented *ugba***.

**Component (mg/100 g)**	**Unfermented ugba**	**Fermented ugba**
**MINERALS**		
Phosphorus	172	–
Calcium	192	110
Iron	16	3.3
**VITAMINS**		
Thiamin	0.07	0.07
Riboflavin	0.32	0.30
Niacin	0.90	0.30

*Source: Duke (1981)*.

**Table 5:** **Changes in mineral contents of African oil bean seeds during processing (mg/kg dry wt)**.

**Mineral**	**Raw**	**Cooked**	**Fermented**	**Canned**
P	351.89 ± 2.58	317.92 ± 2.24	291.02 ± 0.53	176.06 ± 12.69
K	127.19 ± 7.99	175.80 ± 12.46	110.39 ± 6.18	156.67 ± 11.49
Na	184.98 ± 12.31	113.49 ± 2.17	172.06 ± 9.42	168.57 ± 7.30
Ca	314.30 ± 11.32	329.29 ± 11.35	208.92 ± 14.37	404.54 ± 13.34
Mg	292.05 ± 9.86	479.37 ± 5.61	334.98 ± 11.07	397.03 ± 2.02
Zn	9.78 ± 0.61	13.47 ± 0.28	9.23 ± 0.78	15.41 ± 1.98
Fe	56.28 ± 5.42	56.80 ± 1.39	42.46 ± 1.02	42.48 ± 3.19
Mn	23.99 ± 3.06	27.71 ± 1.69	26.87 ± 0.36	15.60 ± 2.75

*Source: Enujiugha and Akanbi (2005)*.

## Chemical and biochemical changes associated with fermentation of African oil bean seeds

The major biochemical changes that take place during the fermentation of African oil bean seeds have been shown to be proteolysis. During the process, the protein component of the cotyledons is hydrolyzed to amino acids. *Bacillus* species are the predominant bacteria during fermentation. Protease activity has been shown to rapidly increase from the start of the fermentation period till the end (Odunfa, 1985a).

Another biochemical change that has been shown to occur during the fermentation of oil bean seeds is lipid hydrolysis. Lipids are usually hydrolyzed to fatty acids by lipases. However, though lipids are one of the major components of the oil bean seeds (43–47%), lipolytic activity is reported to be low during the fermentation of the oil bean seeds (Achinewhu, 1986; Njoku and Okemadu, 1989; Onwuliri et al., 2004). Enujiugha (2003) found out that the principal fatty acid of the seeds, linoleic acid, increased from 60.68 to 67.57% of the total fatty acids while oleic acid decreased from 26.95 to 22.59% during fermentation.

Carbohydrates constitute about 4–17% of the total components of the oil bean seed and the major sugars identified in the bean are oligosaccharides hydrolyzed by amylases (Achinewhu, 1982). These are oligosaccharides that are hydrolyzed by amylases to simple sugars during the fermentation process. Monago et al. (2004) observed that the content of this carbohydrate decreased significantly as fermentation time increased.

Obeta (1983) found out that pH increased from 6.5 at 0 h to 9.0 at 48 h and declined to 7.1 at 72 h. The rise in pH has been attributed to the abundant production of ammonia during the fermentation due to protein hydrolysis and deaminase activity.

Also, moisture content has been found to increase throughout the period of fermentation (52–56.90% to 71.20–73%; Odunfa and Oyeyiola, 1985; Njoku and Okemadu, 1989; Ogueke and Aririatu, 2004). The increase in moisture is believed to be due to the hydrolytic activities of the microorganisms. However, Odunfa and Oyeyiola (1985) and Ogueke and Aririatu (2004) believe that the high moisture level brought about by fermentation predisposes the product to rapid spoilage.

## Anti-nutritional content of *Ugba*

The African oil bean seeds are inedible in its unfermented state because it suffers from some drawbacks. Little is known about anti-nutritional factors in the raw and fermented African oil bean seeds. Although, Kar and Okechukwu (1978) and Enujiugha and Agbede (2000) reported the presence of a number of anti-nutritional and /or toxic factors, our recent studies (Table 6), have revealed the detection of tannins, saponins, alkaloids, steroids, glycosides, flavonoids, and phytate in the unfermented African oil bean seed (Okorie and Olasupo, 2014). This study also showed that processing and fermentation drastically reduced the content of these toxic factors in the fermented product (Table 7) (Okorie and Olasupo, 2014), mainly due to soaking of the seeds overnight and washing in water before fermentation. This had a significant effect on all the phytochemicals/anti-nutritional factors identified. Tannin was reduced from 12.58 to 3. 65 mg/100 g, saponin from 52.00 to 22.00 mg/100 g, phytate from 25.63 to 14.47 mg/100 g, glycosides from 34.76 to 11.33 mg/100 g, alkaloids from 2.52 to 0.14 mg/100 g, flavonoids from 4.66 to 2.49 mg/100 g and steroids from 26.48 to 5.43 mg/100 g. Alkaloids and tannins were completely removed from the samples after 24 and 48 h of fermentation respectively.

**Table 6:** **Preliminary assay for anti-nutritional factors and phytochemicals in African oil bean seed (Okorie and Olasupo, 2014)**.

**Phytochemical**	**Processing method**		**Fermentation period (h)**		
	**Unsoaked**	**Soaked**	**24**	**48**	**72**
Tannin	+++	+	−	−	−
Saponin	+++	++	+	+	+
Flavonoid	+++	+	+	+	+
Alkaloid	++	−	−	−	−
Steroid	++	+	+	+	+
Glycoside	+++	+	++	+	+

*+++, very high; ++, high; +, low; −, absent*.

**Table 7:** **Effect of soaking and fermentation period on the anti-nutritional/phytochemical contents of African oil bean seed**.

**Phytochemical (mg/100 g)**	**Soaking period (h)**					**Fermentation period (h)**			
	**0**	**6**	**12**	**18**	**24**	**0**	**24**	**48**	**72**
Tannin	12.58	10.26	7.02	4.63	3.65	3.65	1.79	0.46	0.00
Saponin	52.00	49.56	40.23	34.29	22.00	22.00	16.06	8.00	2.00
Flavonoid	4.66	4.02	3.46	2.96	2.49	2.49	1.96	1.10	0.43
Alkaloid	2.52	1.94	1.03	0.76	0.14	0.14	0.06	0.00	0.00
Steroid	26.48	12.06	8.68	6.97	5.43	5.43	3.68	2.96	2.07
Glycoside	34.76	30.54	22.09	17.78	11.33	11.33	8.64	5.71	0.78
Phytate	25.63	22.06	18.34	15.69	14.47	14.47	8.67	1.26	0.15

*Source: Okorie and Olasupo (2014)*.

## Microbiological safety of fermented African oil bean seeds

Most works on African fermented foods (*ugba* inclusive) have centered on the isolation and characterizations of organisms involved in the fermentation processes. Not much effort seems to have been made toward the occurrence and growth of possible pathogens in the product. However, Adewunmi et al. (2014) used a combination of genome-based culture dependent and independent techniques to examine *iru* microbiota and reported bacterial species with both spoilage and pathogenic history. In addition, genome typing of *Bacillus* species isolated from *okpehe* and *soumbala* identified species of *Bacillus cereus* with enterotoxin production potential (Ouaba et al., 2008; Oguntoyinbo et al., 2010). It is therefore very important to use genotypic method in combination with phenotypic data to assess microbial quality of fermenting *ugba*, in order to guarantee its microbial safety. Furthermore, because of the stress associated with the food processing, it would be important to use culture dependent and independent methods in order to find/detect non-culturable or not yet cultured microorganisms. Available information in literature shows that organisms such as *E. coli, Staphylococcus aureus* and other members of the *Enterobacteriaceae* have been isolated from condiments in West Africa (Isu and Njoku, 1997; Okorie and Olasupo, 2013a).

## Selection of starter cultures for controlled fermentation of *Ugba*

The traditional method of production of *ugba* involves natural solid state fermentation of the African oil bean seeds. This chanced inoculation method has the inherent drawback of possible growth and occurrence of pathogens in the final product. Although, microbiota that best adapted brings about the final product, variation in final product due to fermentation time and unhygienic handling does affect the product and its consistency.

Selection and application of starter cultures in the production process has been identified as critical to the elimination of pathogens and spoilage microbes (Holzapfel, 2002). Several efforts have been made on the selection and application of starter cultures in a controlled fermentation of some fermented condiments including *ugba* in Nigeria. Oguntoyinbo et al. (2007) used a combination of highly proteolytic and bacteriocin producing starter cultures for the production of *okpehe*, a fermented *Prosopis africana* cotyledon. Isu and Ofuya (2000) studied the use of pure cultures of *Bacillus subtili*s attached to cowpea and maize granules in the fermentation process of *ugba*. They monitored changes in pH, amino-nitrogen and protease activity as fermentation indicators, carried out with the immobilized cells. Protease activity increased from 4.5 to 27.65 mg N/min for the immobilized cells with respect to 10.5 mg N/min produced by the natural fermentation, and there was a reduction in the fermentation time to 48 h as compared to 96 h for the natural fermentation process.

Okorie and Olasupo (2013b) developed controlled fermentation of *ugba* using *B. subtilis* and *B. lichenformis* singly and as mixed cultures fermentation. The process fermentation time was reduced from 96 to 48 h. *Ugba* produced with the starters were similar in terms of color, taste and nutritional content to those produced by natural fermentation.

Several other attempts have been made to control the fermentation of this product with similar results as stated above (Ogueke and Aririatu, 2004; Eze et al., 2014). There, however, still exists a need for more field application and extension of starter cultures to small and cottage processors of condiments in Nigeria.

## Conclusion

*Ugba* is an important part of the diet of the Ibos and other ethnic groups in the eastern and southeastern parts of Nigeria. It is produced through a natural solid state fermentation of the oil bean seeds. The major microorganisms involved in the process are *Bacillus* species. These microorganisms metabolize the protein content of the seeds into free amino acids and ammonia, having undergone a biochemical reaction during the fermentation process known as proteolysis.

Fermentation of the oil bean seeds leads to increase in the nutritional values of the product. The natural process of its production, and the subsistent level at which the condiment is being produced leaves the safety of this product in doubt and makes its quality inconsistent. Efforts at controlled fermentation of the product have shown that some of these observed drawbacks could be overcome by the application of starter cultures in the production process. There is therefore a need to make the local processors of this product realize the potential benefits derivable from the application of starter cultures in their process line.

## Author contributions

NO: Participated in conception, literature search, design and write up. CO: Participate in literature search, design and write up. FO: Participated in literature search, design and write up.

### Conflict of interest statement

The authors declare that the research was conducted in the absence of any commercial or financial relationships that could be construed as a potential conflict of interest.
